# IFN- alpha blocks IL-17 production by peripheral blood mononuclear cells in patients with chronic active hepatitis B Infection

**DOI:** 10.1186/1471-2334-14-55

**Published:** 2014-02-01

**Authors:** Fang Cui, Jiangping Meng, Peng Luo, Pu Chen

**Affiliations:** 1 Department of Laboratory Medicine, The First Affiliated Hospital of Chongqing Medical University, No. 1 Youyi Road, Chongqing, Yuzhong District, China; 2 Department of Obstetrics and Gynecology, Assisited Reproductive Center, The First Affiliated Hospital of Chongqing Medical University, Chongqing 400016, China

**Keywords:** Chronic hepatitis B infection, IFN-α, IL-17, IL-10

## Abstract

**Background:**

IFN-α has been used to treat patients with chronic active hepatitis B (CAHB). Recent studies have implicated the IL-23/Th-17 pathway in the pathogenesis of CAHB. In this study, we investigated whether IFN-α could affect this pathway.

**Methods:**

Peripheral blood mononuclear cells (PBMCs) obtained from patients with active CAHB (n = 61) and controls (n = 32) were cultured with or without IFN-α, and the levels of IL-17 and IL-10 in the supernatants were determined by ELISA, while the frequency of IL-17-expressing cells was measured by FACS. Similar experiments were also conducted with isolated CD4+ T cells from controls. Furthermore, an experiment using an anti-IL-10 antibody was performed to examine the underlying mechanisms of action of IFN-α.

**Results:**

Both the levels of IL-17 and the frequency of IL-17-expressing cells were significantly higher in the PBMCs from CAHB patients than in the controls. IFN-α significantly decreased IL-17 production and the frequency of IL-17-expressing cells in PBMCs from both patients and controls. On the other hand, IFN-α increased IL-10 production by PBMCs from patients and controls. Anti-IL-10 antibody was able to neutralize the inhibitory effect of IFN-α on IL-17 production by PBMCs.

**Conclusions:**

In vitro experiments showed that IFN-α could inhibit IL-17 expression and increase IL-10 production by PBMCs and CD4+ T cells. The inhibitory role of IFN-α on IL-17 production was partly mediated by IL-10.

## Background

Hepatitis B virus (HBV) infection, the major cause of chronic liver injury and inflammation, can lead to liver fibrosis, cirrhosis and hepatocellular carcinoma [1,2]. IFN-α has been used for the treatment of HBV infection-associated complications with promising results. The mechanisms whereby IFN-α exerts its immunomodulatory effects in HBV-infected patients are, however, not completely understood.

It was suggested that the IL-23/Th-17 pathway is implicated in the pathogenesis of the immune response to promote liver damage [3,4], and that the effective treatment of CAHB patients with IFN-α might be mediated via this pathway. Recent studies have shown that Type I IFNs, including IFN-α, could inhibit IL-17 production by peripheral blood mononuclear cells (PBMCs) [5], and Type I IFNs were able to stimulate IL-10 production by activated CD4^+^ T cells [6]. IL-10 is a cytokine of T-regulatory Type 1 cells that can inhibit the production of IFN-γ [7] and antigen presentation [8]. It has also been reported that IL-10 can restrain the pathogenic role of Th17 cells [9]. However, it is not yet known whether IFN-α can inhibit IL-17 in CAHB, and, if so, whether it inhibits IL-17 production directly or indirectly (for instance, through the regulation of IL-10 expression). This study was designed to investigate whether IFN-α could inhibit IL-17 production by PBMCs in CAHB patients and to elucidate the mechanism that is involved in this process. Our results showed that IFN-α could significantly inhibit IL-17 production in association with increased IL-10 production by PBMCs from both CAHB patients and controls. Furthermore, similar findings were confirmed using isolated CD4^+^ T cells from controls. Interestingly, we found that the inhibitory role of IFN-α on IL-17 production was partially mediated by IL-10.

## Methods

### Study population

Sixty-one inpatients with CAHB were enrolled in the study (38 men and 23 women with an average age of 38.7 years). The diagnostic and treatment criteria were based on the clinical practice guidelines of the “European Association for the Study of the Liver” [10], and the Asian-Pacific consensus statement on the management of chronic hepatitisB: a 2012 update [11]. For all patients, there was no evidence of metastatic or autoimmune liver disease. The inclusion criteria were: serum HBsAg positive for at least 6 months, alanine aminotransferase (ALT) >2 times of the normal level (< 72 IU), and Log HBV DNA >5, but no evidence for HCC, or concomitant of HCV, HDV, HIV infection and autoimmune liver disease. The excluded criteria were: exposure to nucleos(t)ide analog(s), acute hepatitis A, B, HCV, HDV, or HIV co-infection, and drug-induced acute hepatitis, existence of renal failure, hepatic decompensation or psychiatric disorders, and central nervous system diseases, such as epilepsy, bone marrow or organ transplants, or immunosuppressive, nephrotoxic, or hepatotoxic medications received within 2 months of enrollment. Thirty-two healthy individuals (17 men and 15 women, with an average age of 33.7 years) acted as controls. Patients and controls were included in this study between October 2012 and November 2013. They were mainly from different regions of Southwest China. Written informed consent according to the Declaration of Helsinki was obtained from each individual, and the above protocol was approved by the Clinical Research Ethics Committee of the first affiliated hospital of Chongqing Medical University. There were no significant differences in age and gender proportion between patients and healthy volunteers (Table 1).

**Table 1:** Demographic and clinical characteristics of the subjects enrolled in the study

	**Healthy controls (n = 32)**	**CAHB (n = 61)**
Age, years		
Mean	32.9	38.7
SD	14.2	13.8
Male/female	17/15	38/23
HBsAg	-	+
Log HBV DNA		
Mean	-	6.34
SD	/	1.36
Serum ALT (IU/liter)		
Mean	15	179.4
SD	5.6	33.2

### Cell isolation and culture

PBMCs were isolated from heparinized blood samples by Ficoll–Hypaque density-gradient centrifugation. Peripheral CD4+ T cells of controls were isolated from PBMCs by human CD4 microbeads (Miltenyi Biotec, Palo Alto, CA, USA) according to the manufacturer’s instructions. The purity of CD4+ T cells was detected using FACS Canton II and FACS Diva software (BD Company, Franklin Lakes, NJ, USA) after staining with anti-human CD4 antibodies (BD Company, USA). The result showed that the purity was >92% in each experiment. PBMCs were cultured for 72 h with or without rhIFN-α 2a (PBL Biomedical Lab, Piscataway, NJ, USA), anti-IL-10 (R&D Systems) at a concentration of 1 × 10^6^ cells/ml in combination with anti-CD3 (5 mg/ml) and anti-CD28 anti- bodies (1 mg/ml) (eBioscience, San Diego, CA, USA). CD4+ T cells (1 × 10^6^ cells/ml) were cultured for 72 h with or without rhIFN-α 2a (1 mg/ml) in combination with anti-CD3 (5 mg/ml) and anti-CD28 antibodies (1 mg/ml) (Miltenyi Biotec,Germany). Supernatants from these cell cultures were used for detecting the concentration of IL-17 and IL-10.

### Intracellular cytokine staining

As a longer incubation period was needed to study the influence of IFN-α on the IL-17 production, we first compared the result of 20-h and 6-h incubation with phorbol myristate acetate (PMA) and ionomycin in five samples. Our result showed that there was no significant difference concerning both IL-17 expression and the frequency of live PBMCs between 20-h and 6-h incubation. Therefore, a 20-h incubation was used in the following experiments. Briefly, PBMCs from patients and controls were cultured with or without rhIFN-α 2a (PBL Biomedical Lab, Piscataway, NJ, USA), at a concentration of 2 × 10^6^ cells/ml in combination with 20 ng/ml PMA and 1 μg/ml ionomycin (Sigma, Saint Louis, MO, USA), for 20 h and then blocked with 10 μg/ml brefeldin A (Sigma, St Louis, MO, USA) for 4 h. The cultured cells were stained with anti-CD3, anti-CD4 antibodies (BD Company, USA), washed, fixed, permeabilized, and subsequently stained with anti-IL-17A (BD Company, USA) or appropriate isotypes (eBioscience, San Diego, CA). FACS analysis was performed using FACS Canton II and FACS Diva software (BD Company, USA) to determine the in vitro effect of IFN-α on the expression of IL-17.

### ELISA

Peripheral blood samples of patients and normal controls were collected and placed in tubes free of pyrogen and endotoxin. After centrifugation at a speed of 1000 × *g* for 15 min, serum samples were harvested and stored at -70°C until use. The concentration of IL-17 and IL-10 in the supernatants of cultured PBMCs and CD4^+^ T cells was measured using the human Duoset ELISA development kit (R&D systems), which has a detection limit of 15 and 31 pg/ml, respectively.

### Statistical analysis

Paired-sample *t*-test was applied for within group comparison and one-way ANOVA was applied for between group comparison using SPSS10.0 software (SPSS Inc., Chicago, IL, USA). Data are expressed as mean ± SEM. p < 0.05 was considered as statistically significant.

## Results

### Concentration of IL-17 in the supernatants is higher in the cultured PBMCs from CAHB patients than in the controls and in both addition of rhIFN-α decreases IL-17 production

**Figure 1:**
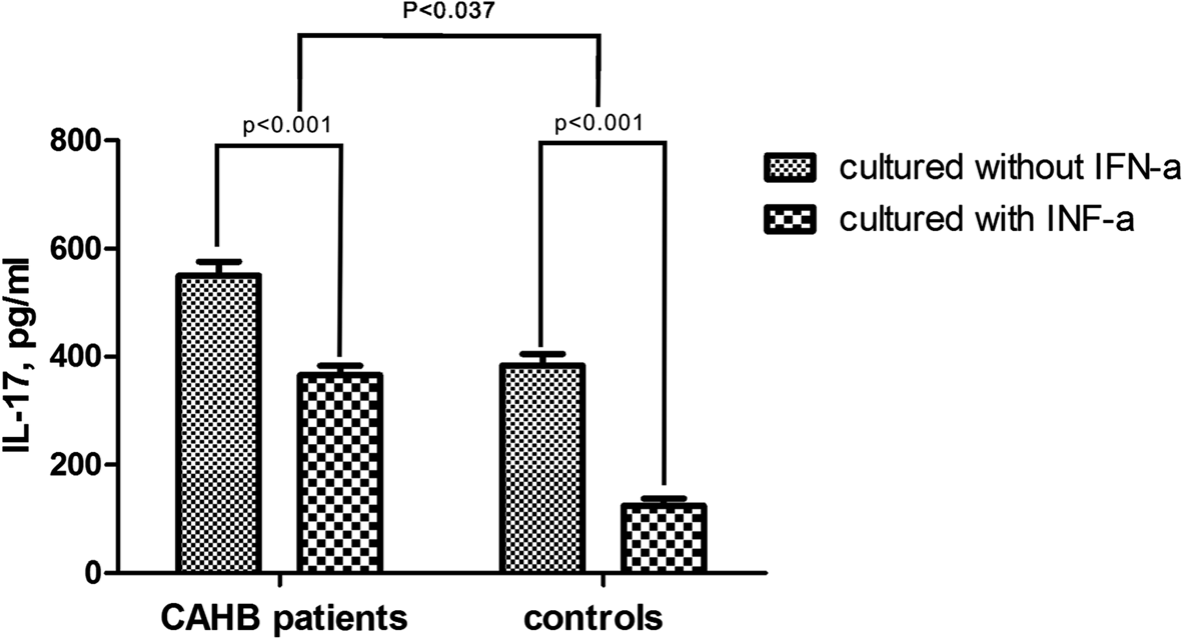
**The inhibitory effect of rhIFN-α 2a on IL-17 production from PBMCs from CAHB patients (n = 61) and healthy controls (n = 32).** PBMCs from CAHB patients (n = 61) and healthy controls (n = 32) were cultured with or without rhIFN-α 2a at a concentration of 1 × 10^6^ cells/ml in combination with anti-CD3 (5 mg/ml) and anti-CD28 anti- bodies (1 mg/ml) for 72 h. IL-17 production in the supernatant of cultured PBMCs was measured by ELISA. Columns represent meanvalues (mean + SEM).

ELISA was performed to determine IL-17 in the supernatants of stimulated PBMCs from patients and controls. Stimulation of PBMCs with anti-CD3 and anti-CD28 antibodies resulted in significant production of IL-17 (p < 0.001). IL-17 levels in the cell culture supernatants of PBMCs from CAHB patients were significantly higher than those obtained from controls (p < 0.037). Addition of rhIFN-α 2a to this cell culture model revealed a significantly decreased IL-17 production both in CAHB patients and controls (Figure 1).

### The frequency of IL-17-expressing CD4+ T cells is significantly increased in CAHB patients and addition of rhIFN-α 2a decreases the frequency of IL-17 expressing cells from both patients and controls

**Figure 2:**
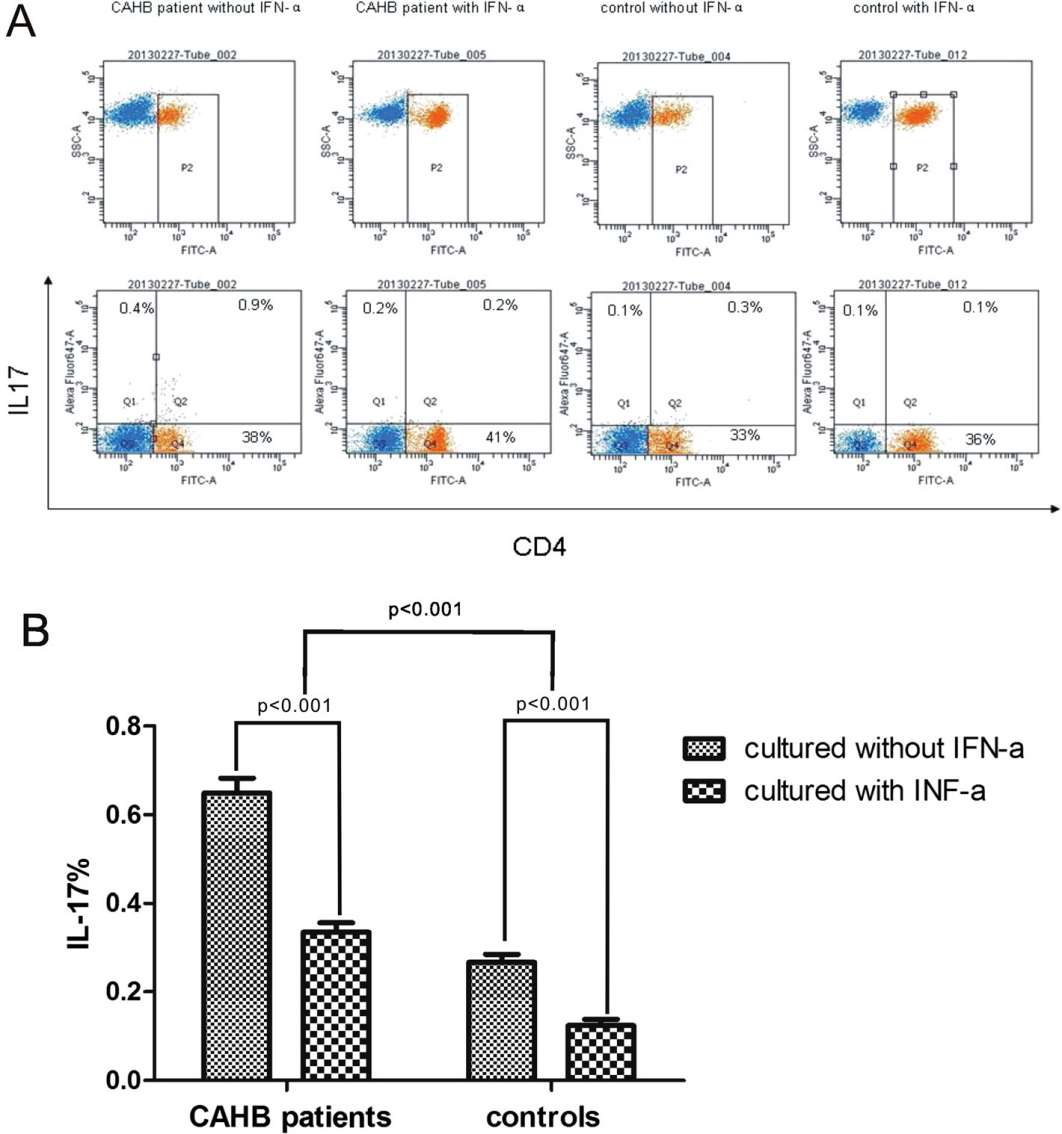
**Detection the Frequencies of IL-17 expressing CD4+ T cells in PBMCs from CAHB patients (n = 61) and healthy controls (n = 32) cultured with or without rhIFN-α 2a by intracellular cytokine staining assay and FACS analysis. (A)** Data from a representative patient. Numbers indicate percentages of positive cells in that quadrant. **(B)** Quantitative analysis of IL-17 -expressing CD4+ T cells. Columns represent mean values (mean + SEM).

We further analyzed the frequency of IL-17- expressing T cells in CAHB patients (n = 61) and controls (n = 32) using FACS analysis. We found that both CD4^+^ and CD4-T cells expressed IL-17, but since the frequencies of IL-17-expressing CD4-T cells were extremely low in CAHB patients, we only investigated the CD4^+^ T cells. We found that frequency of IL-17-expressing CD4^+^ T cells was significantly increased in CAHB patients compared to healthy controls (p < 0.001). Addition of rhIFN-α 2a could decrease the frequencies of IL-17-expressing T cells in CAHB patients and in controls (p < 0.001) (Figure 2).

### rhIFN-α 2a increases IL-10 production by PBMCs from CAHB patients and controls

**Figure 3:**
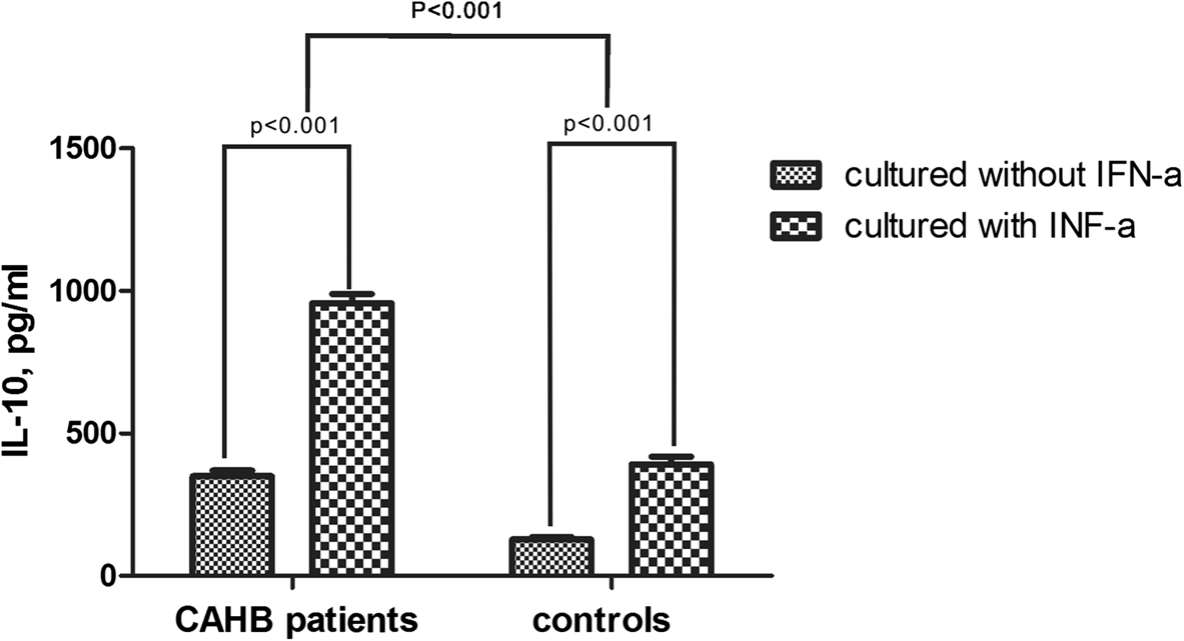
**rhIFN-α 2a increase the production of IL-10 from PBMCs from CAHB patients (n = 61) and healthy controls (n = 32).** PBMCs from CAHB patients (n = 61) and healthy controls (n = 32) were cultured with or without rhIFN-α 2a at a concentration of 1×10^6^ cells/ml in combination with anti-CD3 (5 mg/ml) and anti-CD28 anti- bodies (1 mg/ml) for 72 h. IL-10 production in the supernatant of cultured PBMCs was measured by ELISA. Columns represent meanvalues (mean + SEM).

ELISA was also performed to determine the levels of IL-10 in the supernatants of stimulated PBMCs from patients and controls. IL-10 levels in the cell culture supernatants of PBMCs from CAHB patients were significantly higher than those obtained from controls (p < 0.001). Addition of rhIFN-α 2a to this cell culture model revealed a significant increase of IL-10 production (p < 0.001) (Figure 3).

### rhIFN-α 2a inhibits IL-17 production, but increases IL-10 production by isolated CD4^+^ T cells from healthy controls

**Figure 4:**
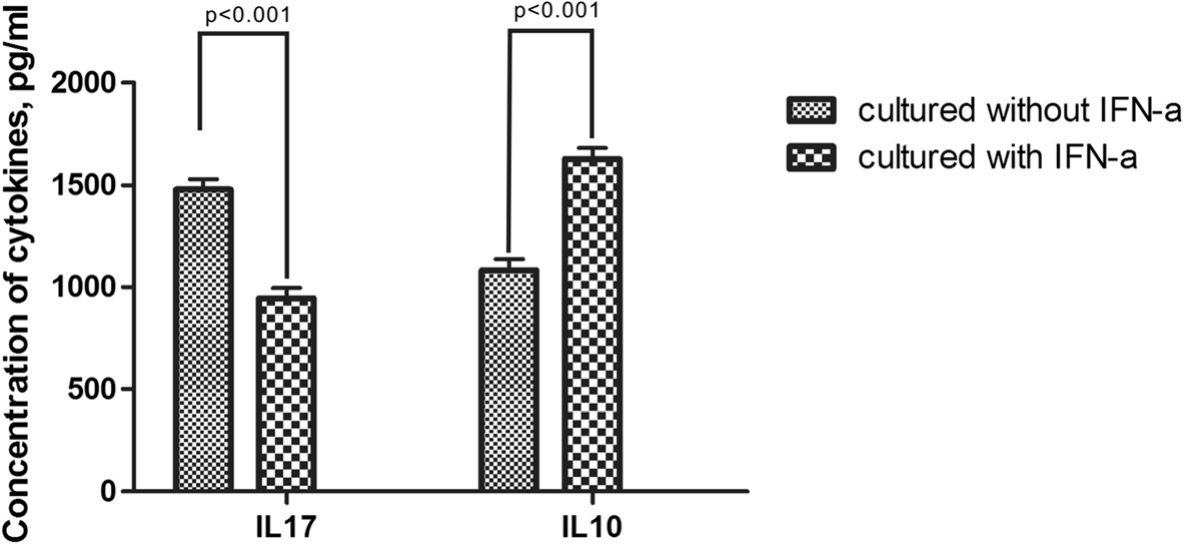
**The Expression of IL-17 and IL-10 in by CD4+ T cells from healthy controls (n =32) cultured with or without rhIFN-α 2a.** CD4+ T cells from healthy controls (n = 32) were cultured with or without rhIFN-α 2a at a concentration of 1x10^6^ cells/ml in combination with anti-CD3 (5 mg/ml) and anti-CD28 anti- bodies (1 mg/ml) for 72 h. IL-10 production in the supernatant of cultured PBMCs was measured by ELISA. Columns represent meanvalues (mean + SEM).

Experiments using isolated CD4^+^ T cells from healthy controls showed that rhIFN-α 2a also significantly inhibited IL-17 production (p < 0.001) and promoted IL-10 production (p < 0.001) (Figure 4). IL-17 and IL-10 levels were much higher in experiments using isolated CD4^+^ T cells from healthy individuals as compared with those in PBMCs.

### Anti-IL-10 antibody partially neutralizes the inhibitory effect of rhIFN-α 2a on IL-17 production by PBMCs from CAHB patients

**Figure 5:**
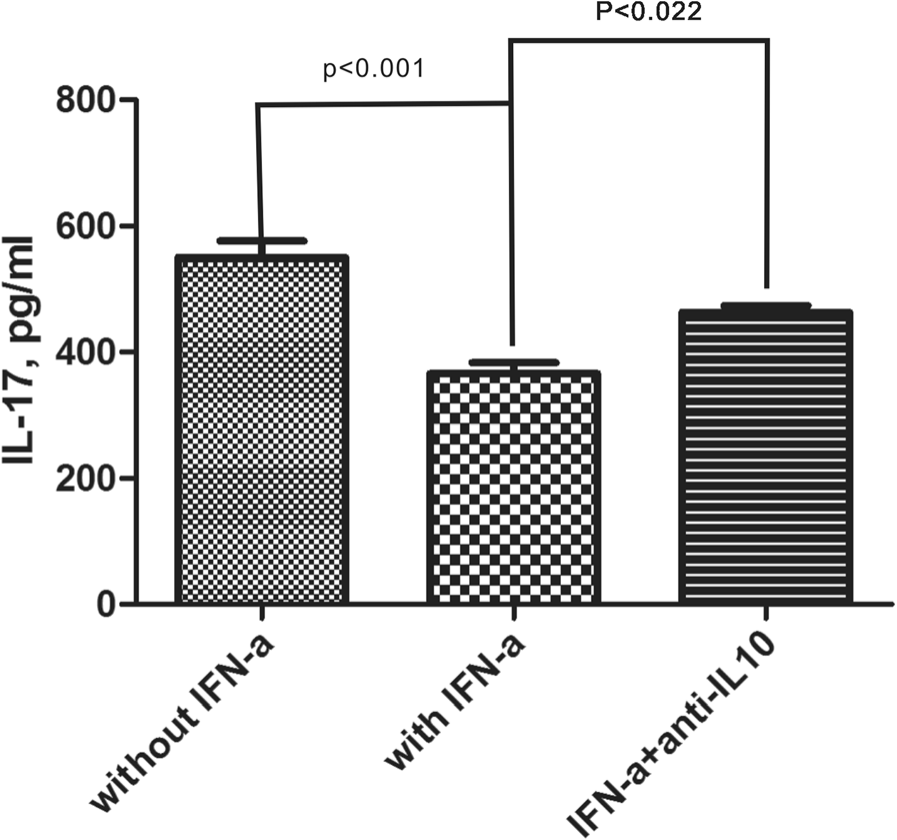
**Anti-IL-10 antibody could partially neutralize the inhibitory effect of rhIFN-α 2a on IL-17 production by PBMCs from CAHB patients (n = 61).** PBMCs from CAHB patients were cultured with or without rhIFN-α 2a, anti-IL-10 antibody in combination with anti-CD3 and anti-CD28 antibodies for 72 h. Concentrations of IL-17 in the supernatants were detected by ELISA. Anti-IL-10 antibody (10 mg/ml) could partially neutralize inhibitory role of rhIFN-α 2a on IL-17 production. Columns represent mean values (mean + SEM).

A significant decrease of IL17 production by PBMCs (p < 0.001) (Figure 1) was observed in the presence of IFN-α, which was associated with an elevated IL-10 level (p < 0.001) (Figure 3). To investigate the role of IL-10, we incubated PBMCs in the presence of IFN-α and an antibody against IL-10. In the presence of this antibody against IL-10, the inhibitory effect of rhIFN-α 2a on IL-17 production was partially blocked (p = 0.022) (Figure 5).

## Discussion

In the present study, IL-17 levels in the cell culture supernatants of PBMCs from CAHB patients were significantly higher than those obtained from controls. Earlier studies showed that the level of IL-17 was elevated in CHB patients with high ALT level and suggested that IL-17 is associated with the active intrahepatic inflammation in CHB patients.

In recent studies of patients with autoimmune hepatitis [12] and hepatitis B [3], the degree of hepatic inflammation was directly correlated with Th17 cell infiltration. In our study, the frequency of peripheral Th17 cells in CAHB patients was found to be significantly higher than that of normal controls. The frequency of Th17 cells in the PBMC population was examined after stimulation with PMA and ionomycin, which are pharmacological T cell-activating agents that mimic signals generated by the T cell receptor (TCR) complex. However, this combinatory stimulation did not replicate the physiological conditions by which T cells are stimulated in vivo.

Our results are also consistent with those reported earlier in CAHB patients. Th17 cells were found to be highly enriched in the peripheral blood and liver, and exhibited a significant potential to exacerbate liver damage during HBV infection [3]. The frequency of peripheral Th17 cells also correlated with ALT, suggesting a potential role for Th17 cells in the immune activation of chronic HBV infection [13]. Recently, it was reported that the frequency of circulating Th17 cells in HBV-infected patients positively correlated with the severity of the disease and the extent of liver injury. Therefore, it has been hypothesized that Th17 cells might contribute to HBV disease progression and development of liver injury [4,14].

IL-10 is an anti-inflammatory cytokine involved in the down-regulation of the immune response. Research has indicated that induction of IL-10 can limit proinflammatory Th17 responses in HBV infected patients [15]. The strong correlation between HBV load and the regulatory T cell (Treg)/Th17 ratio in CAHB indicates that the imbalance of these two cell types might contribute to HBV persistence. Th17-mediated inflammatory diseases [16] strongly support the notion that Th17 cells play a proinflammatory role in the pathogenesis of hepatitis B and HBV infection-related injury.

The IFNs are a family of proteins with an important role in protection against viral infections, tumor growth, inflammation and angiogenesis [17]. The IFNs are divided into three classes whereby the first two are most important from an immunological point of view. In humans, the main Type I IFNs consist of IFN-α (with 13 subtypes) and IFN-β (1 subtype). IFN-α is produced in large amounts by plasmacytoid dendritic cells, whereas IFN-β is mainly produced by fibroblasts.

IFN-α has been successfully used for the treatment of hepatitis [18] and we wondered if IFN-α has any effects on IL-17 and IL-10. In this study, we found that IFN-α 2a could inhibit IL-17 expression and increased IL-10 production by PBMCs from CAHB patients and controls. Further experiments using isolated CD4^+^ T cells from healthy controls showed similar results.

In our CAHB patients, we observed a stronger induction of IL-10 as compared with controls following incubation of PBMCs with IFN-α. This result seems to suggest that a latent negative regulatory mechanism already exists in these patients but that it is not triggered and is insufficient to suppress the overwhelming ongoing Th17 response. We also investigated whether IFN-α could exert its effects through up-regulation of IL-10. The results showed that IFN-α induced both PBMCs and CD4^+^ T cells to produce IL-10. We then questioned whether IFN-α could inhibit IL-17 expression through an up-regulation of IL-10. Therefore, an experiment using an anti-IL-10 antibody was performed to examine underlying mechanisms of action of IFN-α. Interestingly, we did find that the anti-IL-10 antibody blocked the inhibition of IL-17 production promoted by IFN-α. These results suggest that the function of IFN-α is at least partially mediated by IL-10.

Also our results concerning the mechanism of action of IFN-α are consistent with those reported earlier in patients with other diseases. Zhang et al. [19] showed that IFN-α inhibited the differentiation of Th17 cells and production of IL-17 through up-regulating TLR7 expression in dendritic cells from patients with multiple sclerosis. It was reported that treatment with IFN-α could induce an increased frequency of CD4^+^CD25^high^ T cells in PBMCs from MS patients [20]. However, Seya and coworkers [21] found that IFN-α from polyI:C-stimulated dendritic cells (DCs) inhibited the expansion of CD4^+^CD25^+^FoxP3^+^ Tregs. Whether a down-regulatory effect of IFN-α on IL-17 production by T cells, as observed in CAHB, is associated with an increased CD4^+^CD25^+^FoxP3^+^ Tregs needs further studies.

We performed these experiments using PBMCs from healthy control patients and ideally the data should also be confirmed using cells obtained from CAHB patients. Other cytokines such as IL-27 that are known to be induced by IFN-α and that can block the differentiation of Th precursor cells into Th17 cells may also be involved but were not included in our study [22,23]. Our experiments using isolated CD4^+^ T cells from healthy controls showed levels of IL-17 and IL-10 in the culture supernatants much higher than in experiments with the same number of PBMCs. The absolute number of CD4^+^ T cells was much higher in such cultures and indicated that these cells are probably the main source of IL-17 and IL-10 in this in vitro model. On the other hand, δγ T cells have also been shown to produce IL-17 and may play an important role. Further studies are needed to establish which T-cell subpopulations are subject to IFN-α regulation.

## Conclusions

In conclusion, our study showed that IFN-α could down-regulate IL-17 levels in association with an up-regulation of IL-10 in CAHB patients and healthy controls. The inhibitory effect of IFN-α on IL-17 production was partially mediated by IL-10. Further studies are needed to establish which T-cell subpopulations are subject to IFN-α treatment.

## Competing interests

The authors declare that they have no competing interests.

## Authors’ contributions

All authors participated in the design, implementation, analysis and/or interpretation of the study. FC and JM led the clinical team at Department of Laboratory Medicine, and were involved in all phases of the study. PL and PC conducted all ELSA test. FC conducted FACS test and the statistical analysis. All the authors revised the manuscript critically for important intellectual content and approved the final version before submission.

## Pre-publication history

The pre-publication history for this paper can be accessed here:

http://www.biomedcentral.com/1471-2334/14/55/prepub
